# Neuronal p38α mediates age‐associated neural stem cell exhaustion and cognitive decline

**DOI:** 10.1111/acel.13044

**Published:** 2019-09-27

**Authors:** Leire Moreno‐Cugnon, Miren Revuelta, Olatz Arrizabalaga, Sandra Colie, Manuel Moreno‐Valladares, Daniel Jimenez‐Blasco, Francisco Gil‐Bea, Irantzu Llarena, Juan Pedro Bolaños, Angel R. Nebreda, Ander Matheu

**Affiliations:** ^1^ Cellular oncology group Biodonostia Institute San Sebastian Spain; ^2^ Institute for Research in Biomedicine (IRB Barcelona) Barcelona Institute of Science and Technology Barcelona Spain; ^3^ Institute of Functional Biology and Genomics (IBFG) Universidad de Salamanca CSIC Salamanca Spain; ^4^ Neurosciences Area Biodonostia Institute San Sebastián Spain; ^5^ CIBERNED Madrid Spain; ^6^ Optical Spectroscopy Platform CIC biomaGUNE San Sebastian Spain; ^7^ CIBERfes Madrid Spain; ^8^ Institució Catalana de Recerca i Estudis Avançats (ICREA) Barcelona Spain; ^9^ IKERBASQUE Basque Foundation for Science Bilbao Spain; ^10^ Present address: Department for Neonatology Charité University Medical Center Berlin Germany

**Keywords:** aging, cognitive decline, neural stem cells, neuronal activity, p38MAPK

## Abstract

Neuronal activity regulates cognition and neural stem cell (NSC) function. The molecular pathways limiting neuronal activity during aging remain largely unknown. In this work, we show that p38MAPK activity increases in neurons with age. By using mice expressing *p38α‐lox* and *CamkII‐Cre* alleles (*p38α∆‐N*), we demonstrate that genetic deletion of *p38α* in neurons suffices to reduce age‐associated elevation of p38MAPK activity, neuronal loss and cognitive decline. Moreover, aged *p38α∆‐N* mice present elevated numbers of NSCs in the hippocampus and the subventricular zone. These results reveal novel roles for neuronal p38MAPK in age‐associated NSC exhaustion and cognitive decline.

## 1 INTRODUCTION

Neurogenesis occurs in the subgranular zone of the dentate gyrus (DG) in the hippocampus and the subventricular zone (SVZ) of the lateral ventricle in the adult mammalian brain. Adult hippocampal neurogenesis arises from neural stem cells (NSCs) within the DG. NSCs give rise to intermediate progenitor cells, which divide generating immature neurons that subsequently integrate into the local neural network as granule cells. Accumulating evidence suggests that adult‐born neurons may play distinct physiological roles in hippocampus‐dependent functions such as memory encoding and mood regulation (Goncalves, Schafer, & Gage, 2016). Age induces a decline in adult NSC activity and neuronal plasticity, which could partially explain some age‐related cognitive deficit symptoms (Capilla‐Gonzalez, Herranz‐Perez, & Garcia‐Verdugo, 2015; Encinas et al., 2011; Goncalves et al., 2016). Neuronal loss or dysfunction also contributes to the onset of age‐related neurodegenerative pathologies.

Increasing evidence reveals that NSC activity is regulated by intrinsic and extrinsic factors. Among the latter, it has been recently shown that neuronal activity controls NSC quiescence and subsequently neurogenesis in the hippocampus (Song et al., 2012; Yeh et al., 2018). The molecular mechanism by which neuronal activity contributes to the regulation of NSCs, and whether this decreases with aging, remains unknown.

p38 mitogen‐activated protein kinase (p38MAPK) is an important sensor of intrinsic and extrinsic stresses and consequently controls key processes of mammalian cell homeostasis such as self‐renewal, differentiation, proliferation and death (Cuadrado & Nebreda, 2010). In the brain, p38MAPK signalling is activated during neurodegenerative diseases and in response to brain injury (Hensley et al., 1999; Irving, Barone, Reith, Hadingham, & Parsons, 2000). Its genetic or pharmacological inhibition ameliorates symptoms of neurodegenerative diseases and protects against ischemia (Barone et al., 2001; Colie et al., 2017; Roy et al., 2015). There is evidence that p38MAPK can regulate some functions in neurons and NSCs during embryo development and postnatal stages (Cheng, Chan, Milhavet, Wang, & Mattson, 2001; Hamanoue et al., 2016; Kummer, Rao, & Heidenreich, 1997). The p38MAPK family comprises four members, with p38α and p38β being expressed at high levels in the brain. p38α has been involved in inflammation and environmental stresses, and there is evidence implicating p38α in neuronal function and cognitive activity with contradictory results (Cortez et al., 2017; Kase, Otsu, Shimazaki, & Okano, 2019; Stefanoska et al., 2018; Xing, Bachstetter, & Eldik, 2015). In this study, we characterized the impact of genetic inactivation of *p38α* specifically in neurons during physiological aging.

## 2 RESULTS

### 2.1 p38MAPK activity in neurons increases with aging

**Figure 1:**
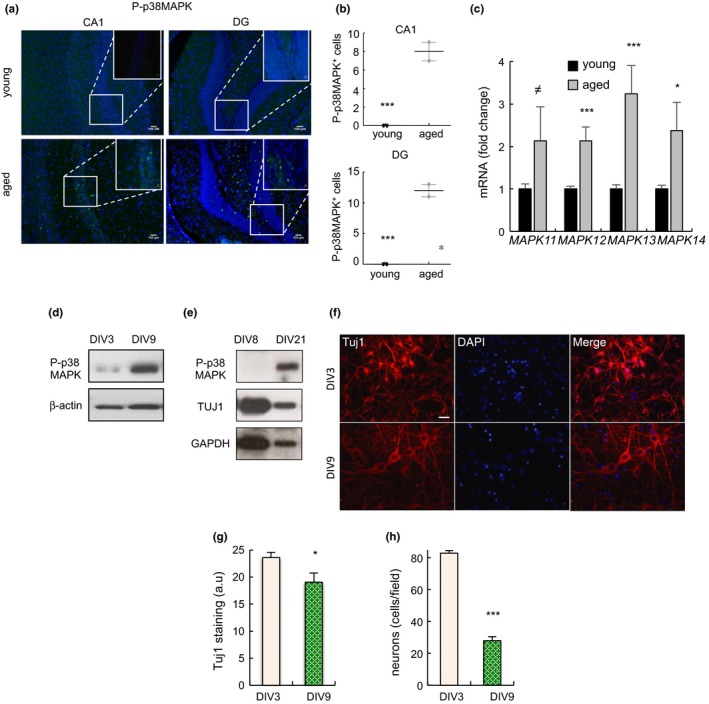
Increased p38MAPK activity in neurons with aging (a) Representative immunofluorescence for phosphorylated p38MAPK (P‐p38MAPK) in CA1 and DG of young (2‐month‐old) and aged (over 24‐month‐old) *C57BL/6J* mice (*n* = 2). (b) Quantification of P‐p38MAPK in these regions. (c) *MAPK11, MAPK12, MAPK13* and *MAPK14* mRNA levels in hippocampus of young and aged *C57BL/6J* mice (*n* ≥ 4). (d) P‐p38MAPK in DIV3 and DIV9 neurons harvested from cortex of *C57BL/6J* embryos (*n* = 3). (e) P‐p38MAPK and Tuj1 in DIV8 and DIV21 neurons harvested from hippocampus from *C57BL/6J* embryos. Results representative of two independent experiments. (f) Representative immunofluorescence of Tuj1 staining and morphology of neurons at DIV3 and DIV9. (g) Quantification of TUJ1 staining at indicated time points. (h) Quantification of numbers of cells at the indicated time points relative to DAPI staining (*n* = 3)

We first determined the activity of p38MAPK in CA1 and DG regions of hippocampus in young (2‐month‐old) and aged (≥2‐year‐old) *C57BL/6J* mice. Immunofluorescence showed that p38MAPK phosphorylated in the activating residues (P‐p38MAPK) was absent or very low in the cells along the CA1 and DG from young mice but significantly increased in over 2‐year‐old animals (Figure 1a,b). Moreover, the expression of all p38MAPK family members (*MAPK11 *(β), *MAPK12* (γ), *MAPK13* (δ) and *MAPK14 *(α)) was elevated in ex vivo analysis using hippocampal tissue obtained from aged mice (Figure 1c).

Long‐term cultured neurons in vitro share multiple characteristics of physiological aging (Lesuisse & Martin, 2002). We cultured neurons harvested from cortex and hippocampus of mouse embryos and observed that cells maintained for longer periods (DIV9 and DIV21 vs. DIV3 and DIV8, respectively) contained higher levels of P‐p38MAPK (Figure 1d,e). Moreover, the elevated P‐p38MAPK in neurons correlated with lower expression of the Tuj1 neuronal marker, loss of synapses and dendritic spines, and enhanced neuronal loss (Figure 1f–h), all characteristics of neuronal aging. These results reveal that the activity of p38MAPK increases with age in neurons in vivo and in vitro.

### 2.2 Genetic deletion of p38α in neurons prevents age‐associated neuronal loss and neuroinflammation

**Figure 2:**
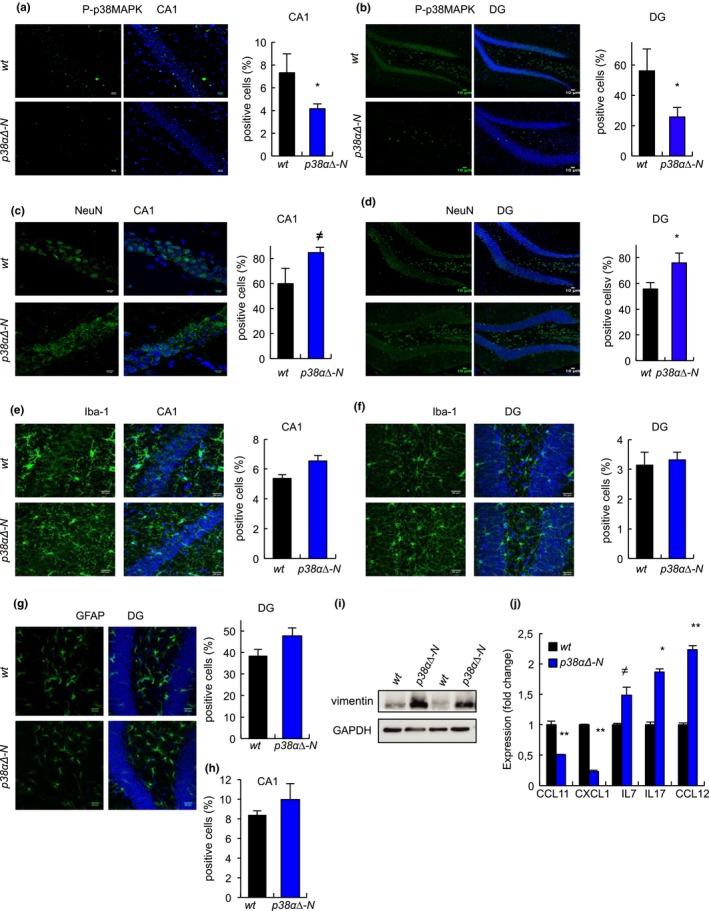
Genetic deletion of *p38α* in neurons prevents age‐associated neuronal loss (a, b) Representative immunofluorescence and quantification for phosphorylated p38MAPK (P‐p38MAPK) relative to DAPI in (a) CA1 and (b) DG of aged (over 24‐month‐old) *wt* and *p38α∆‐N* mice (*n* ≥ 4). (c, d) Immunofluorescence and quantification for NeuN^+^ cells in (c) CA1 and (d) DG of aged *wt* and *p38α∆‐N* mice (*n* ≥ 4). (e, f) Immunofluorescence and quantification for Iba‐1^+^ cells in (e) CA1 and (f) DG of aged *wt* and *p38α∆‐N* mice (*n* ≥ 4). (e, f). (g, h) Immunofluorescence and quantification for GFAP^+^ cells in (g) CA1 and (h) DG of aged *wt* and *p38α∆‐N* mice (*n* ≥ 4). (i). Immunoblot of vimentin from ex vivo hippocampal tissue from *wt* and *p38α∆‐N* aged mice (*n* = 2). (j) Quantification of differentially expressed cytokines in ex vivo hippocampal tissue from aged *wt* and *p38α∆* mice (*n* = 2)

To characterize the function of p38MAPK in neuronal aging in vivo, mice expressing *p38α‐lox* and *CamkII‐Cre* alleles (*p38α∆‐N*), which specifically downregulate p38α in neurons (Colie et al., 2017), were maintained for more than 2 years. Immunostaining analysis revealed reduced P‐p38MAPK^+^ cells in the CA1 (4.15 ± 1.38 vs. 7.31 ± 0.44) and DG (25.71 ± 18.23 vs. 56.06 ± 6.97) of *p38α∆‐N* mice that were over 24‐month‐old compared to *wt* control (expressing p38α) mice of the same age (Figure 2a,b). This indicates that p38α is responsible for ≈50% of the p38MAPK activity in aged neurons. In this context, the number of cells expressing the mature neuron marker NeuN was increased in both regions by ≈40%, particularly in the DG of *p38α∆‐N* mice (Figure 2c,d).

p38MAPK is an important regulator of the central nervous system inflammatory responses (Bachstetter & Van Eldik, 2010). To determine the effect of deletion of neuronal *p38α* on neuroinflammation, we first measured the expression of Iba‐1 and GFAP, markers of microglia and astrocytes, respectively. Immunofluorescence showed no statistically significant differences in the number of Iba‐1^+^ and GFAP^+^ cells in the CA1 and DG areas between *p38α∆‐N* and *wt* mice (Figure 2e–h and Figure S1), although *p38α∆‐N* mice presented slightly higher GFAP^+^ cells in both regions, which correlated with increased levels of vimentin (Figure 2i). Next, we used an array to measure the expression of a battery of 40 cytokines in the hippocampus of old mice, but no differences were detected in key pro‐inflammatory cytokines such as TNF*α*, IFNγ, IL6 or IL1*α* between *p38α∆‐N* and *wt* mice. However, we found some cytokines and chemokines differentially expressed, including CCL11 and CXCL1 that were reduced, and IL7, IL17 and CCL12 increased in *p38α∆‐N* mice compared with *wt* mice (Figure 2j). These data indicate that reduction in p38MAPK activity alters the expression of several cytokines and chemokines in the hippocampus.

### 2.3 Genetic deletion of p38α in neurons prevents age‐associated NSC exhaustion

**Figure 3:**
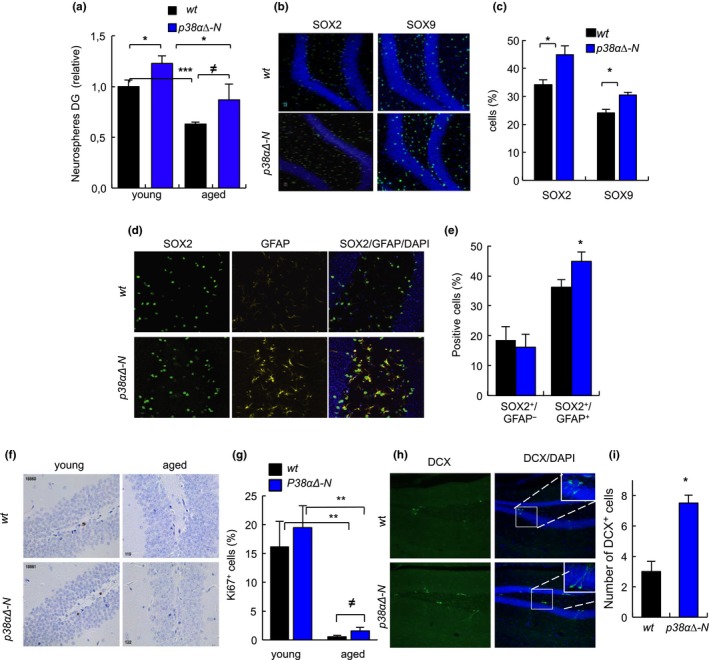
Genetic deletion of p38α in neurons prevents NSC exhaustion in the hippocampus (a) Quantification of neurospheres from DG of *wt* and *p38α∆‐N* mice (*n* = 3). (b, c) Representative immunofluorescence and quantification of SOX2 and SOX9 in the DG of aged mice (*n* ≥ 4). (d, e) Representative images and quantification of SOX2 (green) and GFAP (yellow) in DG of aged mice (*n* ≥ 6). (f, g) Representative images and quantification of Ki67 immunohistochemistry in the DG of young (2‐month‐old) and aged (over 24‐month‐old) *wt* and *p38α∆‐N* mice (*n* ≥ 3). (h, i) Representative images and quantification of DCX‐positive cells in the DG of aged mice (*n* ≥ 6)

Next, we characterized the population of NSCs in the hippocampus. First, we observed that the number of DG cells capable of forming neurospheres in vitro was ≈30% higher in *p38α∆‐N* than in *wt* mice at different ages (Figure 3a). Importantly, these results were corroborated in vivo where the number of SOX2^+^ and SOX9^+^ cells, markers of quiescent NSCs (Hutton & Pevny, 2011; Scott et al., 2010; Shin et al., 2015), was elevated by 30% and 24%, respectively, in *p38α∆‐N* mice that were over 24‐month‐old (Figure 3b,c). In line with this, NSCs expressing both SOX2 and GFAP were also higher by 23% in *p38α∆‐N* mice (Figure 3d,e), whereas the numbers of intermediate progenitors SOX2^+^ and GFAP^‐^ were similar between *p38α∆‐N* and *wt* mice (Figure 3d,e). Moreover, analysis of Ki67^+^ cells showed a marked decrease in proliferation in both genotypes with age, but still *p38α∆‐N* mice displayed increased proliferation in young (19.5% vs. 16.57%) as well as in advanced age (1.57% vs. 0.55%) (Figure 3f,g). Finally, the staining of DCX, marker of immature neurons, was twofold higher in aged *p38α∆‐N* mice (Figure 3h,i). Together, these data show that genetic deletion of p38α in neurons contributes to maintain NSC quiescence and delays the age‐associated exhaustion of NSCs.

**Figure 4:**
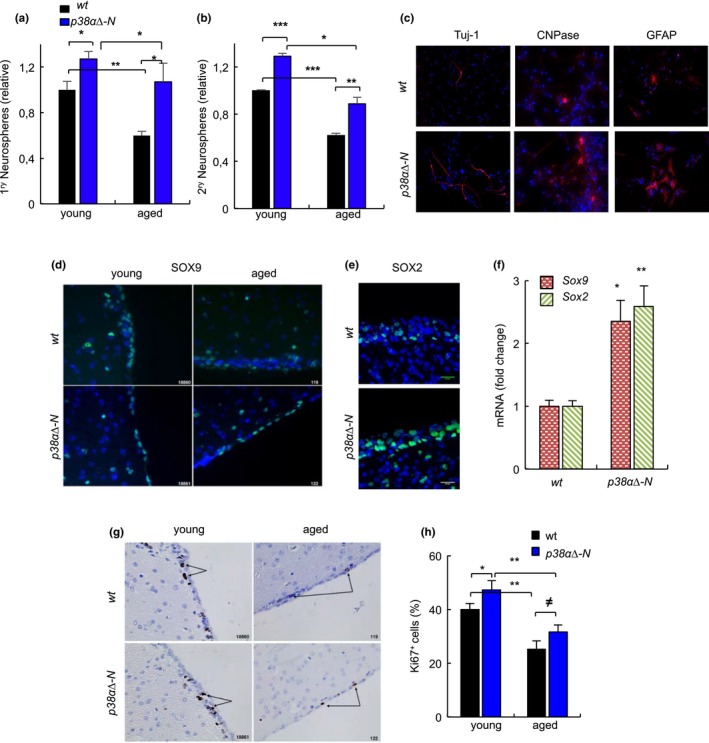
Genetic deletion of p38α in neurons prevents NSC exhaustion in the SVZ (a, b) Quantification of primary and secondary neurospheres from SVZ of *wt* and *p38α∆‐N* mice at different ages (*n* = 6). (c) Representative immunofluorescence of TUJ1, GFAP and CNPase derived from aged neurospheres of *wt* and *p38α∆‐N* mice differentiated in the absence of growth factors and 1% of serum (*n* = 3). (d, e) Representative images of SOX9 and SOX2 NSC markers in the SVZ of *wt* and *p38α∆‐N* mice at indicated ages (*n* ≥ 3). (f) mRNA levels of *Sox2* and *Sox9* in the SVZ of aged *wt* and *p38α∆‐N* mice (*n* = 4). (g, h) Representative images and quantification of Ki67^+^ cells in young and aged *wt* and *p38α∆‐N* mice (*n* ≥ 3)

We also characterized the pool of NSCs in the neurogenic niche of the SVZ. Ex vivo, the ability of SVZ cells to form neurospheres was significantly higher in both young and aged *p38α∆‐N* mice compared with SVZ cells from *wt* mice of similar age (Figure 4a). Same results were obtained counting secondary neurospheres (Figure 4b), indicative of increased self‐renewal ability. This correlated with enhanced differentiation capability, as neurospheres from *p38α∆‐N* mice gave rise to higher numbers of neurons and glial cells, measured by immunofluorescence of TUJ1, CNPase and GFAP (Figure 4c). In vivo, the SVZ of aged p*38α∆‐N* mice displayed increased number of SOX2^+^ and SOX9^+^ cells (Figure 4d,e) as well as higher mRNA levels (≈2 fold increase) compared to the SVZ of *wt* mice of the same age (Figure 4f). Moreover, Ki67 staining showed decreased proliferating cells on both genotypes with aging, but *p38α∆‐N* mice presented a higher number of Ki67^+^ cells (17% in young and 28% in aged) than *wt* mice (Figure 4g,h). Taken together, our results identify a role for neuronal p*38α* in the decline of NSC activity in both DG and SVZ with aging.

### 2.4 Genetic deletion of p38α in neurons delays cognitive decline

**Figure 5:**
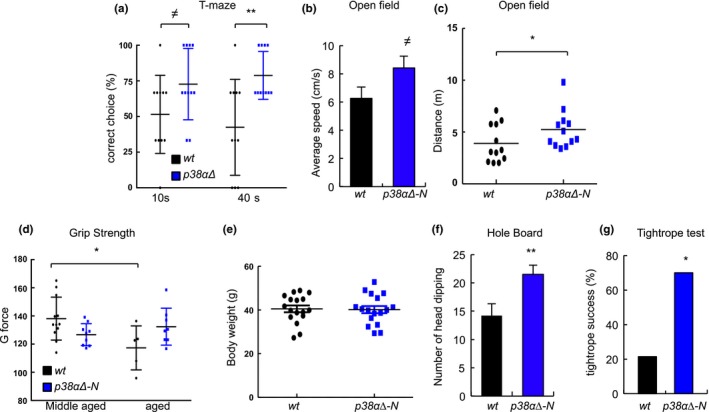
Genetic inactivation of p38α in neurons delays cognitive decline (a) T‐maze correct choice rate of aged (over 16‐month‐old) *wt* and *p38α∆‐N* mice (*n* = 11) with 10‐ and 40‐s retention. (b) Average speed (cm/s) in peripheral zone in *wt* and *p38α∆‐N* mice (*n* ≥ 10) in the open‐field test and (c) individual and average distance (m) performed by *wt* and *p38α∆‐N* mice (*n* ≥ 10) in the open‐field test. (d) Grip strength values obtained in middle age (10‐ to 15‐month‐old) and aged > 15 months *wt* (*n* = 8 and *n* = 5, respectively) and *p38α∆‐N* (*n* = 9 and *n* = 8) mice. (e) Body weight of (over 12‐month‐old) *wt* and *p38α∆‐N* mice (*n* = 17). (f) Quantification of head‐dipping in aged (over 16‐month‐old) *wt* (*n* = 11) and *p38α∆‐N* (*n* = 6) mice. (g) Percentage of aged *wt* (*n* = 14) and *p38α∆‐N* (*n* = 10) mice that successfully passed the tightrope test. Fisher's exact test for each age group is relative to *wt*

Finally, we investigated whether the cellular effects observed upon neuronal deletion of p38α could be translated at the brain functional level. Therefore, we carried out several cognitive and noncognitive tests. First, we performed T‐maze, openfield and novel objective recognition tests, which have been associated with hippocampal activities of memory, learning, locomotion, anxiety and exploratory behaviour, and are known to deteriorate with aging (Gage, Dunnett, & Bjorklund, 1984; Lamberty & Gower, 1992; Shoji, Takao, Hattori, & Miyakawa, 2016). Specifically, when comparing those tests in aged mice, *p38α∆‐N* mice performed better than *wt* mice in T‐maze correct rate, with the correct choice percentages of *p38α∆‐N* mice being significantly higher than those of *wt* mice at 10‐ and 40‐s delay time (Figure 5a). In addition, the time required for correct choice was also reduced in *p38α∆‐N* mice (Figure S2a). We also detected differences in the openfield test, with *p38α∆‐N* mice covering higher distance, with elevated average speed and resting less time in both central and peripheral areas (Figure 5b, c and Figure S2b). In the novel object recognition test, we observed no differences in discrimination index, although *p38α∆‐N* mice spent more time exploring both objects (Figure S2c). In contrast, we did not find differences in grip strength or body weight between aged *p38α∆‐N* mice and *wt* mice (Figure 5d, e), suggesting that the observed phenotypes are mostly dependent on improved cognitive activity rather than motor skills. To further confirm this idea, we performed two additional cognition tests, hole board and tightrope, which are known to deteriorate with aging and, at least the former, measures specific cognitive functions of neophilia and/or anxiety‐like behaviour (Carrasco‐Garcia, Arrizabalaga, Serrano, Lovell‐Badge, & Matheu, 2015; Lamberty & Gower, 1992). Aged *p38α∆‐N* mice displayed increased head‐dipping frequency (21.5 vs. 14.2 in *wt* mice) in the hole‐board test (Figure 5f). Moreover, the tightrope neuromuscular test performance was also significantly improved in aged *p38α∆‐N* mice with 70% succeeding it compared to only 20% of *wt* mice (Figure 5g). These results reveal that inhibition of p38MAPK activity in neurons helps to maintain cognitive activity in aged mice.

## 3 DISCUSSION

Our results show that neuronal p38MAPK activity increases with aging both in neuronal cultures ex vivo and in the hippocampus in vivo, and this elevation is deleterious for neuronal function. Indeed, genetic inactivation of p38α specifically in neurons, using mice expressing *p38α‐lox* and *CamkII‐Cre* alleles, ameliorates age‐associated neuronal loss in the hippocampus. It bears mention that the DG followed by CA1 and CA3 layers of the hippocampus are the brain regions where CaMKIIα is most abundantly expressed (Dragatsis & Zeitlin, 2000; Wang, Zhang, Szabo, & Sun, 2013). In particular, over 70% of granular neurons in the DG and pyramidal neurons in the CA1 and C3, as well as mossy neurons, strongly express CaMKIIα (Wang et al., 2013). Thus, our results show that genetic deletion of *p38α* in the majority of neurons in the hippocampus prevents age‐associated neuronal loss, but we cannot rule out that *p38α* deletion could also take place in additional brain areas where CaMKIIα is expressed with lower intensity (Wang et al., 2013).

Our study also reveals that *p38α∆‐N* mice present elevated cell proliferation levels not only in the DG of the hippocampus but also in the SVZ in young and importantly in aged mice. Moreover, aged *p38α∆‐N* mice maintain higher number of quiescent NSCs as well as of intermediate progenitor cells and neuroblasts. Thus, our data indicate an age‐dependent role for neuronal *p38α* in regulating NSC exhaustion and regenerative decline in the aging brain. This is in line with our in vitro neurosphere studies showing that aged *p38α∆‐N* NSCs maintain enhanced self‐renewal and differentiation potential. It is possible to surmise that age‐associated upregulation of neuronal p38α expression, and hence enhanced p38MAPK activity, induces NSC exhaustion (a) promoting niche deterioration that requires NSC activation and proliferation, and hence their exhaustion at advanced ages, and (b) failing to send proper signals to NSCs, and thereby affecting cell fate decisions. Both options have been described in different contexts (Schultz & Sinclair, 2016).

In support of this idea, we detected differential expression of some cytokines and chemokines in the hippocampus. Specifically, aged *p38α∆‐N* mice displayed higher levels of IL7, a cytokine that promotes neuronal survival (Michaelson, Mehler, Xu, Gross, & Kessler, 1996), and decreased levels of the chemokines CCL11 and CXCL1, whose elevation has been associated with systemic aging, and their high levels are detrimental to neurogenesis and cognitive function, particularly in the case of CCL11 (Villeda et al., 2011; Wolfe, Minogue, Rooney, & Lynch, 2018). Moreover, we also detected higher VIMENTINE levels, which are associated with astrocyte activation and mobilization (Liu et al., 2014; Wilhelmsson et al., 2019). Thus, the positive impact of *p38α* deletion, and consequent reduction in p38MAPK activity, in neurons is likely mediated by controlling the neuroinflammatory status of the niche. This is consistent with the role of p38MAPK pathway, and in particular p38α, as inflammatory mediator in the central nervous system (Bachstetter & Van Eldik, 2010). Future studies should provide additional information on the specific neurons that are responsible for the phenotypes identified in *p38α∆‐N* mice.

We have previously shown that adult *p38α∆‐N* mice are healthy and are indistinguishable from *wt* littermates, also in terms of cognitive behaviour, based on the performance of novel object recognition test (Colie et al., 2017). In advanced age, T‐maze and open‐field tests reveal improved spatial working memory and enhanced locomotor activity in aged *p38α∆‐N* mice. Thus, deletion of *p38α* in neurons is sufficient to delay the age‐associated decline of hippocampal‐specific cognitive activities. In line with this idea, middle‐aged mice systemically expressing a p38α^AF^ dominant‐negative mutant present improved context fear discrimination task, although they do not show improvements in additional cognitive activities such as memory, exploratory behaviour or locomotion (Cortez et al., 2017). Together, these results show the requirement of increasing age for the deleterious action of p38MAPK elevation in brain homeostasis and cognitive activity in the hippocampus. Moreover, the improved performance of *p38α∆‐N* mice in holeboard and neuromuscular coordination tests further highlights the role that the p38MAPK pathway, and p38α in particular, exert in maintaining cognitive functions.

In summary, our results reveal an unprecedented function of p38MAPK regulating neuronal activity to reduce NSC function with aging. Moreover, they support recent studies showing that different neuronal types and neuronal activities control NSC quiescence at adult stage (Song et al., 2012; Yeh et al., 2018) and extend this idea to physiological aging. Ultimately, our data provide experimental evidence supporting the pharmacological targeting of p38MAPK for therapy against age‐associated cognitive decline.

## CONFLICT OF INTEREST

The authors declare no competing financial interests.

## AUTHOR CONTRIBUTIONS

LM‐C, MR, OA and MM‐V performed all the experiments except indicated. IL analyzed confocal images. ARN and SC provided *p38α∆‐N* mice and performed Ki67 immunohistochemistry experiments. JPB, DJ and FG‐B isolated neurons from cortex and hippocampus and carried out experiments with them. JPB and ARN contributed to the experimental design, data analysis and discussion. AM directed the project, contributed to data analysis and wrote the manuscript.
