# Supplementary material for: Neuronal p38α mediates age‐associated neural stem cell exhaustion and cognitive decline
Source: Aging Cell. 2019 Sep 27;18(6):e13044. doi: 10.1111/acel.13044 (PMC6826142; doi:10.1111/acel.13044)
Supplement: Supplementary file 1 [file ACEL-18-e13044-s001.pdf]

MATERIAL AND METHODS

Mice handling and ethics statement

The C57Bl6 (Jackson Laboratory) mice and p38α-floxed mice crossed with B6.Cg-Tg (Camk2a-cre) T29-1Stl/J mice, from now on named *p38αΔ-N*, were housed in specific pathogen-free barrier areas of the Biodonostia Institute and of the Institute for Research in Biomedicine (IRB) respectively. Mice were maintained under a 12-hour light/12 hour dark cycle at 22ºC with controlled humidity and with food and water provided *ad libitum* and handled in compliance with the animal research regulations specified in the European Communities Directive [2010/63/EU]. All Animal studies were approved by the Biodonostia Institute Animal Care Committee.

Tissue immunohistochemistry and immunofluorescence

Mice were perfused with heparinized saline (25 IU/ml) followed by 4% paraformaldehyde (PFA, Sigma). *For immunohistochemistry*, brain was dissected and post-fixed in 4% PFA overnight and then embedded in paraffin. For histological analysis brains were sectioned in 5 µm slides with SM2010 R Sliding Microtome (Leica). Immunostaining was performed for 2 h at room temperature (RT) using antibodies against Ki67 (Novocastra) and secondary HRP conjugated anti-rabbit (ImmunoLogic). Hematoxilin was used as a counterstaining.

For immunofluorescence, coronal serial sections of 50 µm were collected via SM2010 R Sliding Microtome (Leica) and selected brain sections were blocked with 10 % donkey serum and incubated with anti- phosphorylated-p38MAPK (1:200; rabbit, Cell Signalling); anti-SOX9 (1:500; goat, R&D); anti-SOX2 (1:500; goat, Neuromics); anti-GFAP conjugated with Alexa Fluor 555 (1:500; mouse, Sigma); anti-DCX (1:200; goat, Santa Cruz); anti-NeuN (1:500; mouse, millipore); anti-Iba1 (1:200; rabbi, Wako Chemicals) overnight at 4 °C. Nuclei were stained with DAPI staining (1:600; Sigma). Images were acquired with an inverted confocal light scanning microscope (CLSM 510Meta, Zeiss) with a 63x objective (NA 1.3, aprochromat) in sequential mode with 1024 x 1024 scan size. Processing and analysis was performed on the maximal intensity projection of the z-stack, and selection of the area was accomplished using the nucleus positives areas. Fiji public domain and *Image J*, open source softwares were used to quantify automatically the number of positive cells. At least 3 slices per animal were used in all cases.

From selected area (same in all animals) channels were transformed into binary after automatic threshold selection and watershed prior positive nuclei counting. Counting of the total nuclei was achieved using DAPI channel after filtering, threshold application and watersed prior automatic analyze particle applying size range from 20-100 µm. Percentage of positive cells is shown relative to total number of DAPI cells.

RNA analysis

Total RNA was extracted with Trizol (Life Technologies). Reverse transcription was performed using random priming and Superscript Reverse Transcriptase (Life Technologies), according to the manufacturer's guidelines. Quantitative real-time PCR was performed using Absolute SYBR Green mix (Thermo Scientific) in an ABI PRISM 7500 thermocycler (Applied Biosystems).

Western blot analysis

Immunoblots were performed following standard procedures. Equal amounts of protein (20 µg) were separated on 15% SDS polyacrylamide gels and blotted onto nitrocellulose membranes (BioRad). Primary antibodies were phosphorylated-p38MAPK (1:200, Cell Signalling), Vimetin (1:500, Sigma), neuronal class III β-tubulin (Tuj-1, 1:1000, Covance), GAPDH (1:1000, Abcam) and β-actin (1:2000, Sigma). Secondary antibodies were HRP-linked anti-mouse or rabbit (DAKO). Detection was performed by chemiluminescence using ECL (Amersham).

Cytokine array

Cytokine levels were detected from 4 aged mice hippocampus, 2 wt and 2 p38αΔ, using mouse cytokine array´s protocol (R&D Systems, ARY006). Briefly, 300µg of hippocampus lysate were used to study 40 different antibodies in duplicate. Samples were incubated overnight at 4°C with the antibodies, washed and incubated with Streptavidin-horseradish peroxidase for 30 min after the exposure with the chemiluminescent reagent mix. Positive spots were detected by autoradiography according to the manufacture´s instruction and quantified by Image Studio software.

Neuron cultures.

Primary cultures of mice cortical and hippocampal neurons were prepared from fetal 15.5 d in C57BL/6 mice, seeded at 2.0×10^5 cells per cm^2 in different-sized plastic plates coated with poly-D-lysine (10 µg/mL) and incubated in Neurobasal (Life Technologies) media. Cells were incubated at 37 °C in a humidified 5% (vol/vol) CO_2-containing atmosphere. Cortical cells were grown for 3 or 9 days, while hippocampal cells were grown for 8 or 21 days to simulate young and old *in vitro* neurons and the medium was changed every 2 day.

.Neurosphere cultures

Isolation, culture, and assays of NSCs were carried out as previously described (Gomez-Gaviro *et al.* 2012). Briefly, NSCs were isolated from the mouse SVZ and HC and grown for 10 days in DMEM/F12 growth medium (Sigma) in the presence of EGF (20 ng/mL, Sigma) and FGF-2 (20 ng/mL, Gibco). Primary neurospheres after being counted, were treated with accutase (Sigma) for 5 min, mechanically dissociated to a single-cell suspension and re-plated in growth medium containing EGF and FGF for 10 days (secondary neurospheres). For differentiation, NSCs were attached in crystal pretreated for 3 hours with laminin (Sigma) and maintained in culture for 7 days in DMEM/F12 without growth factors.

Cell immunofluorescence

Cells were fixed with 4% paraformaldehyde for 15 minutes and washed with phosphate-buffered saline (PBS) supplemented with 0.5% Triton X-100. Subsequent to blocking with PBS and 10% FBS, cells were incubated with Tuj1 (1:500; Covance); CNPase (1:500; Millipore) and GFAP (1:500; Sigma) antibodies for 1 h. Nuclei were stained with DAPI staining (1:600; Sigma). Images were acquired as in the case of tissue immunofluorescence.

Behavioral studies in mice

<u>T-maze test</u>

The experiment was performed in a wood T-maze with walls of 20 cm high, alleys of 10 cm wide and length of 30cm of the main alley and side alleys. The side alleys were closed off from the main alley by movable doors. A week before habituation, all animals were partially food restricted and maintained until each animal is above 85% of its free-feeding body weight until the day of the testing as previously described (Shoji *et al.* 2012). The food reward was a 5 g food pellet. The full experiment consisted of three parts: habituation, training, and testing. Briefly, during habituation, all animals were placed on the T-maze until they ate a piece of food or 90 s had elapsed. During training, all animals underwent six trials a day. Each trial consisted of two runs: a forced run and a free run. on the forced run, mice were forced to obtain a piece of food from one goal arm of the T-maze, with the other arm blocked and in the free run mice were allowed to choose either goal arm. If the mice chose the arm opposite the one they had been forced into during the forced run, they received the food reward. The training period ended after control animals made 70 % correct choices on two consecutive days. Finally, the testing period consist on three 10 s delay and three 40 s delay trials. Mice were then tested for their performance in the maze recording the number of correct entries (%) and the duration (s).

<u>Novel object recognition test</u>

Animals were first habituated to the experimental room for a 30 min period. On the first day, animals were habituated to the apparatus (15 x 28 x 50 cm) for a 15 min period. On the next two days, animals were allowed to freely explore two identical novel objects for a 15 min period. On the test day, one of the objects was replaced by a new different one and the animal was allowed to freely explore for 15 min. Exploratory behavior was scored for investigation time of each object in the test session. Results were averaged for total exploration time in objects, and the percentage of discrimination index (DI%) that allows discrimination between the novel and familiar objects [DI% = TN /(TN + TF)*100].

<u>Hole board test</u>

The hole-board test was carried out using a grey 40 x 40 cm iron plate with 16 holes, each with a diameter of 3 cm. Each mouse was placed in the center of the apparatus and left to explore the arena for five minutes. The frequency of head dipping into the holes was recorded as a measure of neophobia.

Open field test

The open-field consists of a rectangular container made of dark polyethylene (45 × 45 × 45 cm). The base of the cage is divided by lines in peripheral and central square. Testing was conducted in a silent room with constant light (300 lux). Mice were individually placed in the center of the apparatus to initiate a 15 minutes test session. Each session was recorded with a video camera and directly analyzed with the SMART (Spontaneous Motor Activity Recording & Tracking) v2.5.2.1 software system (Panlab, Barcelona, Spain). Central and peripheral covered distance (m) and velocity (mm/s) were analyzed.

Motor coordination Test (Tightrope test)

Mice were placed on a bar of circular section (60 cm long and 1.5 cm diameter) and test was considered successful when a mouse did not fall during a period of 60 seconds in at least one trial out of two consecutive trials.

Body weight

Animal's body weight was controlled using a balance (TE15025, Sartorius).

Grip Strength

A Grip Strength Meter (Bioseb) was used to measure forelimb grip strength Mice were positioned horizontally in a grip bar and pulled back slowly and steadily until they released their grip. This was repeated five times at one-minute intervals and the peak force in grams was recorded on a digital force transducer. Tension was recorded by the gauge at the time the mouse released its forepaws from the bar. The highest three measurements were used for the analysis of the forelimb grip strength. G force is shown relative to each mice body weight.

Data evaluation

Data are presented as mean values ± S.E.M. with the number of experiments in parentheses (n). Unless otherwise indicated, statistical significance (p-values) was calculated using the Student´s t test. Asterisks (≠, *, **, and ***) indicate statistically significant differences ($p<0.1$, $p < 0.05$, $p < 0.01$, and $p < 0.001$, respectively). The data that support the findings of this study are available from the corresponding author upon reasonable request.

References

Gomez-Gaviro MV, Scott CE, Sesay AK, Matheu A, Booth S, Galichet C, Lovell-Badge R (2012). Betacellulin promotes cell proliferation in the neural stem cell niche and stimulates neurogenesis. *Proc Natl Acad Sci U S A*. **109**, 1317-1322.

Shoji H, Hagihara H, Takao K, Hattori S, Miyakawa T (2012). T-maze forced alternation and left-right discrimination tasks for assessing working and reference memory in mice. *J Vis Exp*.

CA1

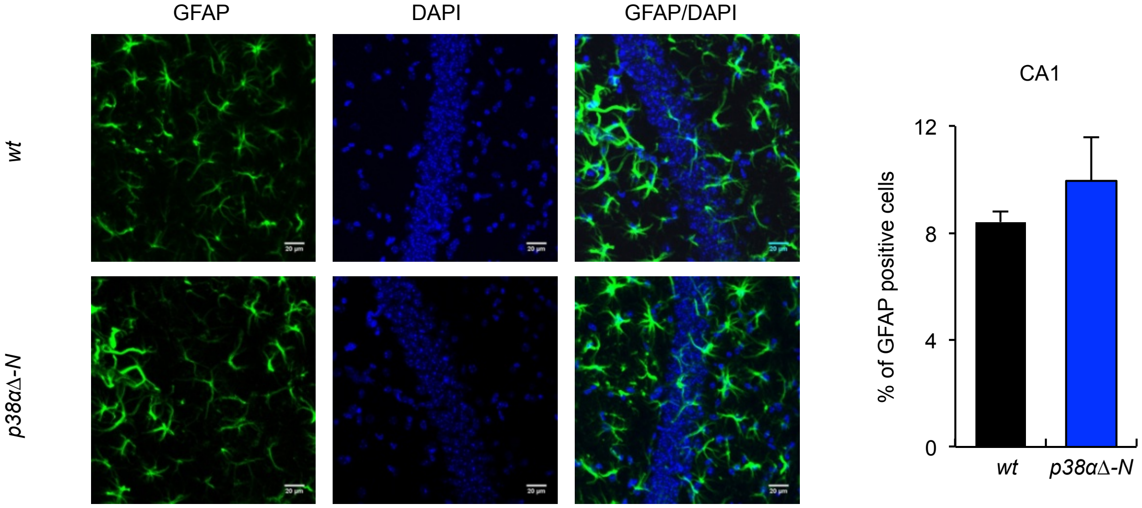

Supplementary Figure 1. Immunostaining of GFAP in aged mice. Representative images and quantification of GFAP immunofluorescence in the CA of *wt* and *p38αΔ-N* mice (n>4). % of positive cells are related to DAPI.

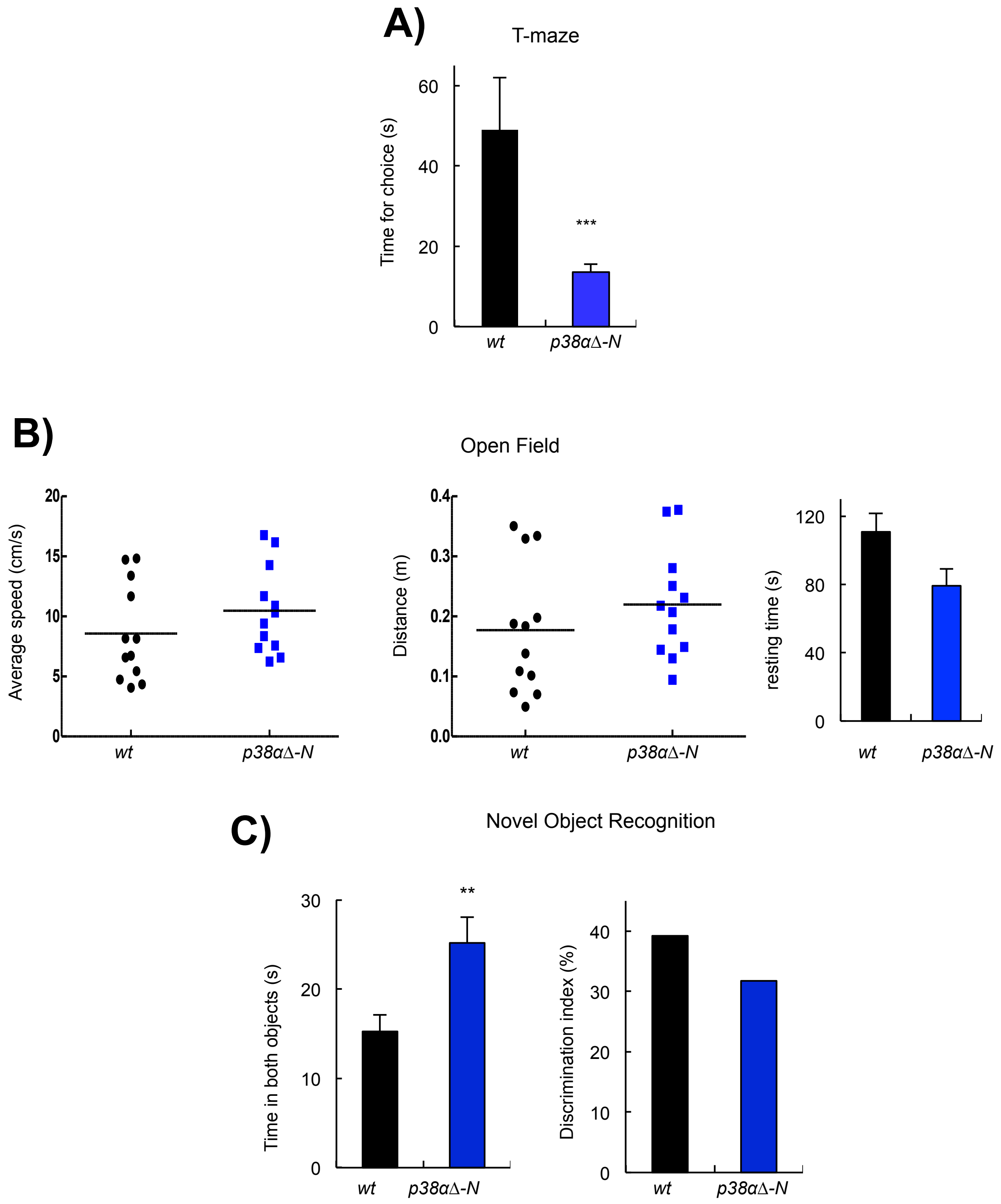

Supplementary Figure 2. Genetic inactivation of *p38α* in neurons delays age-associated cognitive decline. (A) Required time for choice in T-maze of mice with 100% of success (*wt*=2, and *p38αΔ-N*=4). **(B)** Changes in average speed (cm/s) (left) and distance (m) (centre) in the central zone, as well as resting time in periphery (right) in open-field test were analysed in *wt* and *p38αΔ-N* mice (n≥10) . **(C)** Results obtained in Novel Object Recognition Test by aged *wt* (n=13) and *p38αΔ-N* mice (n=12). Total time (seconds, s) spent during the testing phase of the experiment in both objects (left), as well as the discrimination index (%) (right) are presented.
